# Adventures in radiosynthesis of clinical grade [^68^Ga]Ga-DOTA-Siglec-9†

**DOI:** 10.1039/c7ra12423f

**Published:** 2018-02-20

**Authors:** Meeri Käkelä, Pauliina Luoto, Tapio Viljanen, Helena Virtanen, Heidi Liljenbäck, Sirpa Jalkanen, Juhani Knuuti, Anne Roivainen, Xiang-Guo Li

**Affiliations:** Turku PET Centre, University of Turku FI-20521 Turku Finland xiali@utu.fi anne.roivainen@utu.fi; Turku PET Centre, Turku University Hospital FI-20521 Turku Finland; Turku Center for Disease Modeling, University of Turku FI-20014 Turku Finland; MediCity Research Laboratory and Department of Medical Microbiology and Immunology, University of Turku FI-20014 Turku Finland; Turku PET Centre, Åbo Akademi University FI-20521 Turku Finland

## Abstract

We finally managed to establish a protocol for generating Good Manufacturing Practice (GMP)-grade gallium-68-labelled 1,4,7,0-tetraazacyclododecane-1,4,7,10-tetraacetic acid conjugated sialic acid-binding immunoglobulin-like lectin 9 motif containing peptide ([^68^Ga]Ga-DOTA-Siglec-9), the first radiopharmaceutical for positron emission tomography imaging of vascular adhesion protein 1.

## Abstract 2

[^68^Ga]Ga-DOTA-Siglec-9 is the first vascular adhesion protein-1 targeting radiopharmaceutical for positron emission tomography imaging of inflammation, and here we present its long-awaited clinical grade radiosynthesis.

## Graphical abstract

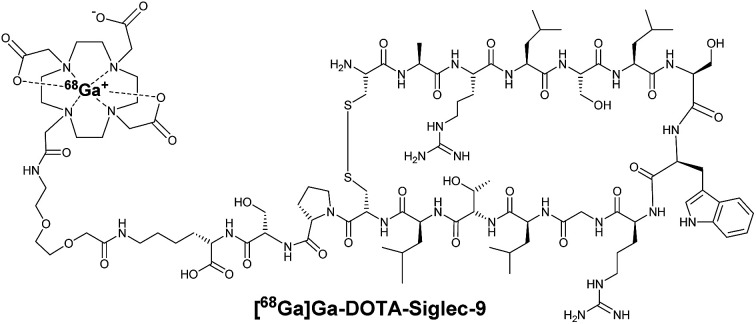

## Introduction

**Scheme 1:**
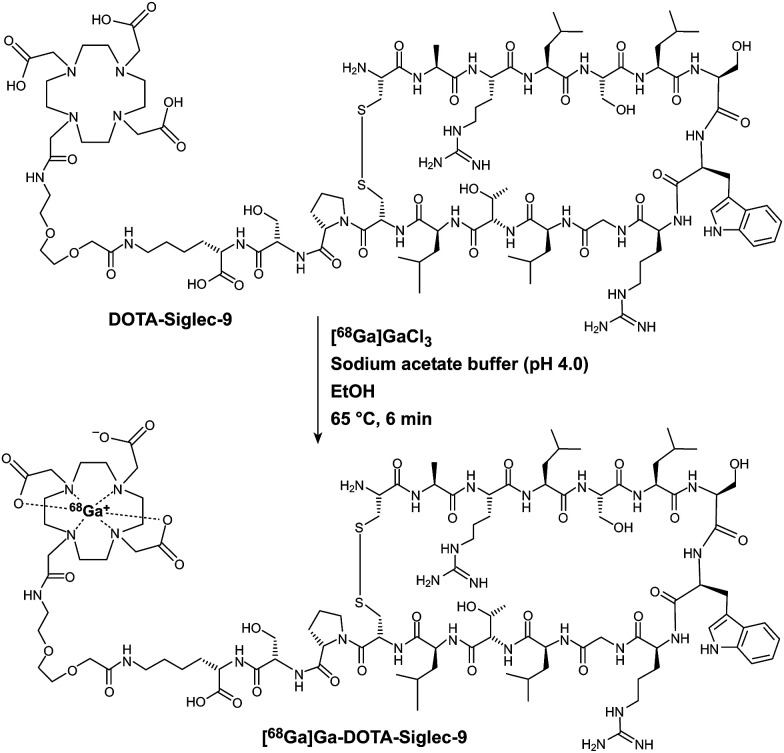
Radiosynthesis of [^68^Ga]Ga-DOTA-Siglec-9.

Vascular adhesion protein 1 (VAP-1) is prominently involved in immune cell trafficking in a number of major human diseases, including rheumatoid arthritis, certain cancers, and atherosclerosis.^1^ VAP-1 was first discovered in inflamed synovial vessels in Turku, Finland, more than two decades ago.^2^ Since then, the international scientific community has devoted a great deal of research effort to VAP-1 targeting. VAP-1 has the unique characteristic of rapidly relocating from intracellular granules onto luminal cell surfaces upon inflammation, making it an ideal target for imaging of inflammation.^1*a*,3^ Accordingly, a few peptide-based ligands and radiolabelled antibodies have been developed in Turku for imaging of VAP-1 using positron emission tomography (PET) techniques.^3,4^ With the aid of phage display and *in silico* modelling methods, a cyclic peptide (sequence CARLSLSWRGLTLCPSK, cysteines forming a disulfide bridge) was identified as a VAP-1 ligand.^3*a*^ This peptide corresponds to amino acid residues 283–297 in the sequence of sialic acid-binding immunoglobulin-like lectin 9 (Siglec-9), and is therefore referred to as the Siglec-9 peptide. We have performed extensive PET studies using Siglec-9 as a lead VAP-1 targeting compound and have modified the peptide with 8-amino-3,6-dioxaoctanoyl spacer, amidation, acylation, glycosylation, or labelled it with various radionuclides.^1*a*,3^ Among the gallium-68 (^68^Ga)-labelled variants, [^68^Ga]Ga-DOTA-Siglec-9 (Scheme 1, DOTA denotes 1,4,7,10-tetraazacyclododecane-1,4,7,10-tetraacetic acid) has been most promising in our preclinical evaluations. [^68^Ga]Ga-DOTA-Siglec-9 enables clear target visualization in a number of disease models in mice, rats, rabbits, and pigs, including synovitis, sterile skin inflammation, atherosclerosis, and acute respiratory distress syndrome.^1*a*,3*a*,5^ Moreover, [^68^Ga]Ga-DOTA-Siglec-9 is also useful for PET imaging of inflammation in infection models, representing a novel direction for its application.^6^

Prompted by the preclinical results, we set out to translate this compound into human clinical use. For this purpose, one prerequisite is the availability of Good Manufacturing Practices (GMP)-grade [^68^Ga]Ga-DOTA-Siglec-9. In general, ^68^Ga-labelling is based on very straightforward chemistry and reaction methods, well-established protocols are available for performing the radiolabelling.^7^^68^Ga radionuclide can be conveniently obtained from a ^68^Ge/^68^Ga-generator rather than requiring an onsite cyclotron. These features facilitate the use of ^68^Ga-labelled compounds in preclinical and clinical research. Indeed, we have established many ^68^Ga-labelled peptides, oligonucleotides, and other molecules in our lab, and a few of them are currently in routine clinical use in our hospital.^8^ However, we encountered many unexpected difficulties in establishing clinical grade [^68^Ga]Ga-DOTA-Siglec-9. In this paper, we report our experiences in developing [^68^Ga]Ga-DOTA-Siglec-9 as a radiopharmaceutical compliant with GMP guidelines.

## Experimental

### General remarks and materials

All radiation work complied with the regulations of the Radiation and Nuclear Safety Authority of Finland (STUK). The GMP-grade precursor compound DOTA-Siglec-9 was prepared by ABX Advanced Biomedical Compounds GmbH (Radeberg, Germany). Other reagents and chemicals were purchased from Sigma-Aldrich-Fluka or Merck. The Modular Lab PharmTracer radiosynthesis device, ^68^Ge/^68^Ga-generator (IGG100-50M, 1.85 GBq, Eckert & Ziegler Isotope Products, Burbank, USA), and synthesis cassettes were from Eckert & Ziegler AG, Berlin, Germany. The Prominence HPLC instrument was Shimadzu (Kyoto, Japan) and the equipped radiodetector was from Ortec (Model 266, Atlanta, GA, USA).

### General procedure for radiosynthesis of GMP-grade [^68^Ga]Ga-DOTA-Siglec-9

The reaction vessel was preloaded with a mixture of sodium acetate buffer, absolute ethanol or ascorbic acid, and precursor DOTA-Siglec-9 (80 μL, 40 μg, 16.5 nmol). [^68^Ga]GaCl_3_ was eluted from the generator with HCl (6 mL, 0.1 M) and loaded onto a strong cation exchange (SCX) or Strata-XC cation exchange cartridge. The [^68^Ga]GaCl_3_ bound to the cartridge was eluted into the reaction vessel with acidified saline or acetone. Before dilution with saline (4 mL, 0.9 mg mL^−1^), the reaction mixture was incubated at different temperatures and for different time durations. The diluted reaction mixture was passed through a solid phase extraction cartridge, which was washed twice with saline (8 mL, 0.9 mg mL^−1^). Purified [^68^Ga]Ga-DOTA-Siglec-9 was eluted into an end product vial with ethanol *via* a filter (0.22 μm) and formulated into physiological saline. An example of optimized radiosynthesis procedure was presented in the ESI.†

## Results and discussion

### Initial radiosynthesis tests

Structurally, [^68^Ga]Ga-DOTA-Siglec-9 consists of a cyclic peptide moiety for VAP-1 targeting and a DOTA chelator for coordination of the ^68^Ga^3+^ ion, connected by a short linker (Scheme 1). We designed the precursor DOTA-Siglec-9, and its GMP grade form was prepared by ABX Advanced Biochemical Compounds GmbH (Radeberg, Germany). To maximize the capacity of a ^68^Ge/^68^Ga-generator, one option is to elute [^68^Ga]GaCl_3_ with large volumes (*e.g.*, 6 mL) of eluent and concentrate the radionuclide onto ion-exchange cartridges. During GMP-grade tracer synthesis, concentrated [^68^Ga]GaCl_3_ is usually eluted out of the ion-exchange cartridges with acidified saline or acetone. However, acetone is not the first choice due to restrictions on its use in humans. In addition, it requires extra steps in the quality control process to determination residual acetone levels. To achieve fully automated production of the radiopharmaceutical, we elected to use the Modular Lab PharmTracer radiosynthesis device and the corresponding cassettes from Eckert & Ziegler AG (Berlin, Germany). Initially, the synthesis steps were designed similarly to the automated radiosynthesis of the ^68^Ga-labelled ligand for targeting prostate-specific membrane antigen (PSMA), and the corresponding PSMA-cassette was used for [^68^Ga]Ga-DOTA-Siglec-9 radiosynthesis. Accordingly, the procedure for radiosynthesis is described above. Along with precursor DOTA-Siglec-9, sodium acetate buffer (0.4 mL, 1 M, pH 4.5) and ascorbic acid (1 mg) were used. The purpose of ascorbic acid is to prevent radiolysis. SCX cartridge was used and the SCX-bound [^68^Ga]GaCl_3_ was eluted into the reaction vessel with acidified saline (5 M NaCl containing 0.14 M HCl). The final pH of the reaction mixture was 3.5–4.0. The reaction mixture was incubated at 100 °C for 15 min and then diluted with saline (4 mL, 0.9 mg mL^−1^). The diluted reaction mixture was passed through a tC18 cartridge and the purified end product was eluted from tC18 with 50% ethanol (2 mL).

### Description of the problems

Using this synthesis method, the decay-corrected radiochemical yield was approximately 30%. Unfortunately, a significant amount (∼30%) of radioactivity remained on the tC18 cartridge, in addition to the unreacted ^68^Ga (∼20%) found in the waste vial; also, some radioactivity (even up to 20%) remained in the reaction vessel. To reduce sticking of the product to the tC18 cartridge we tried increasing the concentration of ethanol. However, even repeated elution (5 × 1 mL) with absolute ethanol did not help. We then replaced the tC18 cartridge with other cartridges (C8, C18, or hydrophilic–lipophilic balance (HLB)), but none improved the outcome.

Even more surprisingly, we did not succeed in quality analysis of the end product from any of the batches with any reversed-phase C18 column fitted to a high-performance liquid chromatography (HPLC) instrument. Instead, the injected radioactivity stuck at the very top of the HPLC column. Subsequently, we modified the HPLC methods in many different ways; for example, changing the flow rates and the properties of solvent and column (*e.g.* C8, C4). To exclude the possibility that sticking was caused by the net positive charge of end product, we tested HPLC columns (*e.g.*, XBridge Shield RP18) with well-blocked silanols. Furthermore, the HPLC instrument (Shimadzu, Prominence, Kyoto, Japan) and the equipped radiodetector (Ortec Model 266, Atlanta, USA) were thoroughly checked and validated, and the analysis was performed on different HPLC workstations. However, nothing helped.

### Strategies in solving the HPLC analysis problems

**Fig. 1:**
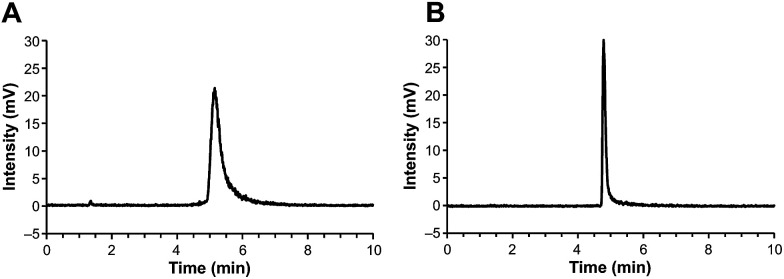
High-performance liquid chromatography (HPLC) analysis of [^68^Ga]Ga-DOTA-Siglec-9, with radioactivity detection, in the presence of (A) 60 μg mL^−1^ or (B) 120 μg mL^−1^ DOTA-Siglec-9 as a carrier. HPLC conditions: Phenomenex Jupiter C18 column, 300 Å, 5 μm, 150 × 4.6 mm. Solvent A was water containing 0.1% trifluoroacetic acid (TFA) and solvent B was acetonitrile containing 0.1% TFA. The conditions were as follows: linear gradient from 23% B to 53% B over 10 min; flow rate pf 1 mL min^−1^. Retention time of [^68^Ga]Ga-DOTA-Siglec-9 was 5.3 min in (A) and 4.9 min in (B).

Previously,^9^ we prepared ^18^F-labelled mannans for preclinical PET applications; mannan is a natural oligosaccharide. Although ^18^F-mannan is not lipophilic, as reflected by its distribution coefficient (log *D* = −1.48) in 1-octanol-phosphate buffered saline (PBS, pH 7.4) at room temperature, it tends to stick to HPLC columns and other surfaces. We noticed that the sticking problem could be solved by increasing the concentration of mannan in the samples. For PET radiopharmaceutical samples, the quantity of analyte, in terms of molarity (or mass), is tiny, which may partially explain why addition of a “carrier” can help analytes travel through the materials packed into HPLC columns. Bearing this in mind, we added different amounts (60–120 μg mL^−1^) of non-radiolabelled GMP-grade precursor compound DOTA-Siglec-9 to the HPLC samples of our [^68^Ga]-labelled end product. Indeed, the radioactivity came out of the HPLC columns. Using this analytical method, the radiochemical purity (>98%) of the end product could be measured. Two examples of the HPLC chromatograms are shown in Fig. 1.

However, another problem remained unsolved. [^68^Ga]Ga-DOTA-Siglec-9 is a totally new radiopharmaceutical that has not previously been studied in humans. Therefore, it is critical to set a dose limit in terms of mass quantity.^10^ Accordingly, we performed extended single-dose acute toxicity studies in rats (unpublished results). We observed no adverse effects; therefore, the concentration limit of the end product was set at 4.1 μg mL^−1^ in a total volume of 10 mL. This limit was derived from the dose limit of another ^68^Ga-labelled DOTA-peptide (bombesin antagonist BAY 86-7548) that has already been established in our hospital.^8*a*^ Unfortunately, due to the need to add a large amount of carrier to the samples for quality control, it was not possible to measure reliably the mass concentration of peptide in the end product when using the carrier-addition method described above. Therefore, to avoid clinical risks, we decided to address the HPLC analysis problem in other ways.

In previous studies, many batches of non-GMP grade [^68^Ga]Ga-DOTA-Siglec-9 were produced in our lab^1*a*,3*a*,5*b*,6,11^ and in our collaborators' labs.^5*a*,11^ The radiochemical purity of non-GMP [^68^Ga]Ga-DOTA-Siglec-9 was measured conveniently by HPLC analysis, although chemical impurities appeared in some batches.^11^ Naturally, non-GMP grade and GMP grade [^68^Ga]Ga-DOTA-Siglec-9 products differ in many ways, including the radiosynthesis processes (full automation *versus* manual synthesis), purification, formulation method, and grade of reagents. In addition to ^68^Ga-labelling of the Siglec-9 peptide, we labelled the same peptide with a prosthetic compound, 5-deoxy-5-[^18^F]fluororibose ([^18^F]FDR),^12^ resulting in the formation of [^18^F]FDR-Siglec-9 suitable for preclinical research.^3*b*^ [^18^F]FDR-Siglec-9 is prepared by conjugating aminooxy-functionalized Siglec-9 with [^18^F]FDR in an anilinium buffer at room temperature; we have never encountered a problem with HPLC analysis of [^18^F]FDR-Siglec-9. Taken together, these experimental results led us to the hypothesis that the sticking problem of GMP-grade [^68^Ga]Ga-DOTA-Siglec-9 was caused by conformational changes/alterations in the peptide structure during radiosynthesis. Specifically, the combination of high salt content (up to 3.5 M NaCl and 0.3 M sodium acetate), high temperature (100 °C), and prolonged reaction time (15 min) may denature the peptide in some way. Accordingly, we reduced the concentration of sodium chloride from 5 M to 1.5 M (the minimum applicable concentration for elution of an SCX cartridge in an experimental setting), decreased the concentration of sodium acetate buffer to 0.08 M, shortened the reaction time to 5 min, and used a lower temperature (*e.g.*, 50 °C). Following these modifications, we found that less radioactivity stuck to solid-phase extraction cartridges and reaction vessels; however, there was no improvement in the HPLC analysis. Meanwhile, another issue arose: at reduced temperatures and with shortened reaction times, we observed increased levels of free ^68^Ga in the waste and the labelling efficiency got worse.

### Radiosynthesis with acetone-elution method

To further reduce the salt content of the reaction mixtures and the temperature of the labelling reactions without compromising the radiochemical yield, we decided to test radiosynthesis with an acetone-based method performed using C4-GA68-PP cassettes from Eckert & Ziegler AG. In this method, [^68^Ga]GaCl_3_ eluate from the generator was passed through a Strata-XC cation exchange cartridge and acidified acetone (0.8 mL, containing 0.02 M HCl and 3.25% water) was used to elute the radionuclide from the Strata-XC into the reaction vessel. The reaction vessel was pre-loaded with a mixture of DOTA-Siglec-9 (40 μg), sodium acetate buffer (2 mL, 0.2 M, pH 4.0) and absolute ethanol (0.2 mL). The reaction mixture was kept at 65 °C for 6 min. During the reaction, acetone was gradually evaporated into the waste *via* vent tubing. After quenching with 4 mL physiological saline, purification with a tC18 cartridge, and formulation (see the details in ESI†), we obtained [^68^Ga]Ga-DOTA-Siglec-9 in high radiochemical yield (90.5%, decay-corrected). As an example, in one of the batches the radioactivity of the end product was 1106 MBq in 10 mL of physiological saline at end of synthesis, and the total synthesis time was 25 min (from generator elution until the end product was ready for administration to humans). The specific (radio)activity was 67 GBq μmol^−1^. The amounts of radioactivity stuck on the tC18 cartridge (14 MBq) or reaction vessel (8 MBq) were insignificant. To make sure that the acetone content in the end product was under the limit (0.5%) required for human use, the automated process was programmed to wash the tC18 cartridges twice during the purification process. In randomly selected batches (*n* = 3), gas chromatography using the standard protocols for our GMP production unit revealed no detectable amount (0.0%) of acetone in the end product; the ethanol content (8.8% ± 0.1%, *n* = 3) was under the limit (10%) as well.

### Optimizing HPLC analysis method

**Fig. 2:**
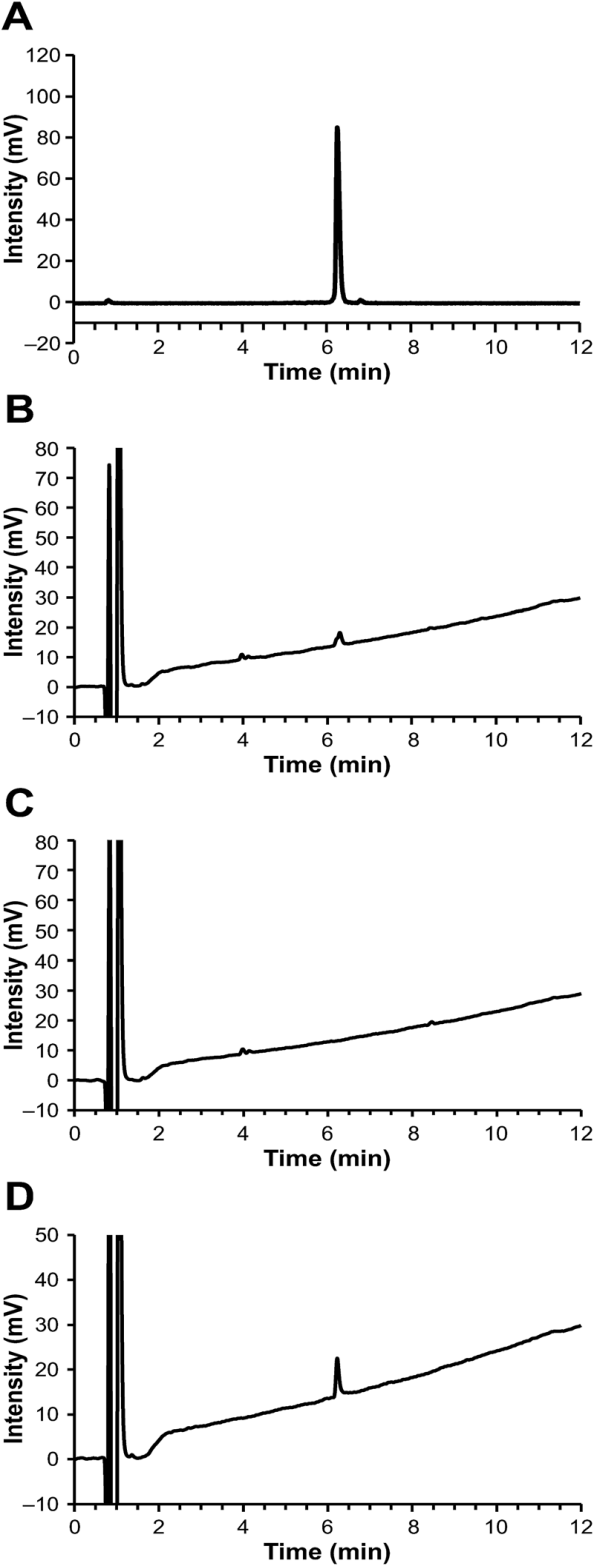
HPLC chromatograms. (A) Radioactivity of [^68^Ga]Ga-DOTA-Siglec-9. (B) The corresponding UV peak of the [^68^Ga]Ga-DOTA-Siglec-9 sample. The peak is small due to the high specific (radio)activity (67 GBq μmol^−1^) at the end of synthesis. (C) UV detection of a control sample obtained from a radiosynthesis without addition of precursor DOTA-Siglec-9. (D) UV detection of DOTA-Siglec-9, as a reference sample. HPLC conditions: Phenomenex Kinetex C18 column, 100 Å, 2.6 μm, 75 × 4.6 mm. Solvent A was water containing 0.16% TFA and solvent B was acetonitrile containing 0.16% TFA. The conditions were as follows: linear gradient from 18% B to 50% B over 12 min; flow rate of 1 mL min^−1^; wavelength at 220 nm for UV detection. Retention time, 6.3 min.

**Fig. 3:**
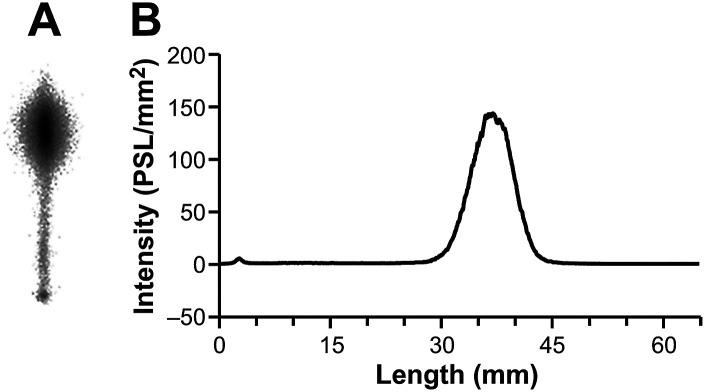
ITLC analysis of [^68^Ga]Ga-DOTA-Siglec-9. The eluent used for iTLC development was 50% methanol in ammonium acetate buffer (pH 3.5, 1 M). (A) Photostimulated luminescence (PSL) imaging of the iTLC plate. During 35 min of iTLC development, the retention factor (*R*_f_) of ^68^Ga-Siglec-9 was 0.57. (B) ITLC profile, analyzed using the Aida image analysis software.

Notably, the [^68^Ga]Ga-DOTA-Siglec-9 produced by this acetone-based method could be analyzed successfully by HPLC, even using an ordinary C18 column (*e.g.* Phenomenex Kinetex reversed phase C18), without a requirement for steep elution gradients (Fig. 2 and ESI Fig. S1†). The radiochemical purity was >98% (*n* = 5). During the variation tests for the HPLC methods, we observed that the concentration of trifluoroacetic acid (TFA) in the eluents played an important role in the analysis. For HPLC analysis of peptide-based PET radiopharmaceuticals, a typical TFA concentration is 0.10% (by volume), but that was not sufficient for [^68^Ga]Ga-DOTA-Siglec-9. At a higher TFA concentration (*e.g.* 0.16% or 0.20%), the peaks were sharp without tailing (Fig. 2 and ESI Fig. S2†). To confirm that no radioactivity was stuck on the column, after analysis we immediately monitored the HPLC column with a well-type gamma-counter (Veenstra Instruments, VDC404, Joure, The Netherlands) and found that only 0.6% of the injected radioactivity was retained. The radiochemical purity was further confirmed by instant thin layer chromatography (iTLC, Fig. 3). Using an eluent consisting of 50% methanol in ammonium acetate buffer (1 M, pH 3.5), [^68^Ga]Ga-DOTA-Siglec-9 migrated nicely up from the baseline on iTLC plates (Varian Inc., Lake Forest, USA), and the purity was >95% (*n* = 3).

Using this HPLC protocol, the chemical purity of [^68^Ga]Ga-DOTA-Siglec-9 was conveniently measured by ultraviolet detection (UV) at a wavelength of 220 nm. The chemical purity was >95% (*n* = 5), and the amount of peptide was below the limit of 4.1 μg mL^−1^. In the UV trace of the HPLC chromatograms, two minor peaks at a retention time of approximately 4 min were observed in every batch product, but not in the blank or reference samples. We performed control experiments to confirm that these impurity peaks were derived from the radiosynthesis system rather than from the peptide itself. Specifically, we performed a control radiosynthesis without the precursor compound DOTA-Siglec-9, but otherwise using exactly the same protocol as for [^68^Ga]Ga-DOTA-Siglec-9. The “end product” solution was subjected to HPLC analysis and two minor peaks appeared at the expected retention times (Fig. 2C).

### PET studies

**Fig. 4:**
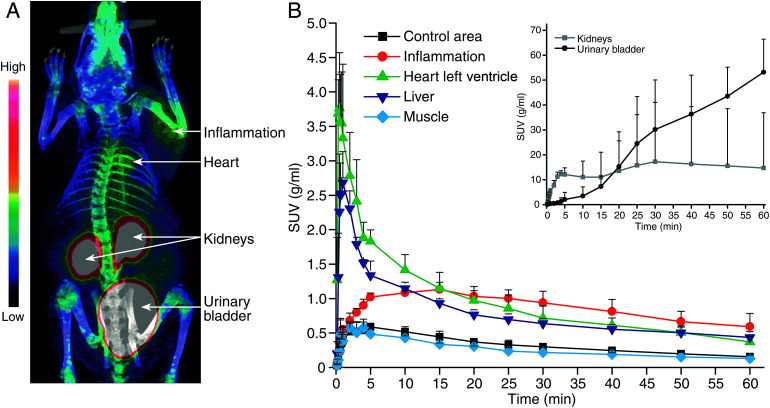
(A) A representative coronal PET/CT image and (B) time-activity curves with the GMP grade [^68^Ga]Ga-DOTA-Siglec-9 in rats with skin-inflammation.

All animal experiments were approved by the National Animal Experiment Board in Finland, and the Regional State Administrative Agency for Southern Finland, and were carried out in compliance with the European Union directive. Previously, we have used a turpentine-induced sterile inflammation model in rats for preclinical evaluation for VAP-1 targeting PET tracers.^3^ We have, therefore, performed *in vivo* and *ex vivo* studies with the GMP grade [^68^Ga]Ga-DOTA-Siglec-9 in the same disease model in rats (*n* = 3). The skin inflammation was induced with turpentine on the right side of the shoulder 24 hours before PET imaging, and the left side was used as control (ESI†). The rats were PET/CT imaged for 60 min in a dynamic mode and the inflamed foci were clearly visualized in each and all the rats (Fig. 4, ESI Fig. S3†). The biodistribution was further confirmed by *ex vivo* radioactivity measurements of excised tissues (ESI, Table S1†).

## Conclusions

In conclusion, we found out that although the DOTA-Siglec-9 peptide is not large, it is sensitive to radiolabelling conditions, *e.g.*, a combination of relatively high salt content and reaction temperature. The key problem we encountered was the sticking of the peptide to the synthesis system (*i.e.*, the reaction vessels and solid-phase extraction cartridges) and HPLC columns. However, this tendency is not a property of [^68^Ga]Ga-DOTA-Siglec-9 itself; rather, it was likely due to denaturation of the peptide during the radiosynthesis process. Ultimately, using an acetone protocol, we successfully established a fully automated protocol for production of GMP-grade [^68^Ga]Ga-DOTA-Siglec-9 with high radiochemical yield and high radiochemical and chemical purity. Notably, this labelling approach achieved high specific (radio)activity. The methods and problem-solving strategies described herein should be useful when radiolabelling other sensitive peptide- or protein-based pharmaceuticals.

## Conflicts of interest

S. J. owns stocks in Faron Pharmaceuticals Ltd. No other authors have any conflicts of interest to declare.

## Supplementary Material

RA-008-C7RA12423F-s001
