# Supplementary material for: Adventures in radiosynthesis of clinical grade [68Ga]Ga-DOTA-Siglec-9
Source: RSC Adv. 2018 Feb 20;8(15):8051–6. doi: 10.1039/c7ra12423f (PMC9078465; doi:10.1039/c7ra12423f)
Supplement: RA-008-C7RA12423F-s001 [file RA-008-C7RA12423F-s001.pdf]

Electronic Supplementary Material (ESI) for RSC Advances.
This journal is © The Royal Society of Chemistry 2018

RSC Advances

Electronic Supplementary Information (ESI)

Adventures in radiosynthesis of clinical grade [^{68}Ga]Ga-DOTA-Siglec-9

Meeri Käkelä,[a] Pauliina Luoto,[b] Tapio Viljanen,[a] Helena Virtanen,[a] Heidi Liljenbäck,[a,c] Sirpa Jalkanen,[d] Juhani Knuuti,[a,b] Anne Roivainen,*[a,b,c], Xiang-Guo Li*[a,e]

[a] Turku PET Centre, University of Turku, FI-20521 Turku, Finland.
[b] Turku PET Centre, Turku University Hospital, FI-20521 Turku, Finland.
[c] Turku Center for Disease Modeling, University of Turku, FI-20014 Turku, Finland
[d] MediCity Research Laboratory and Department of Medical Microbiology and Immunology, University of Turku, FI-20014 Turku, Finland.
[e] Turku PET Centre, Åbo Akademi University, FI-20521 Turku, Finland.

E-mails: xiali@utu.fi (X.G.L.) and anne.roivainen@utu.fi (A.R.)

Radiosynthesis of GMP-grade [^{68}Ga]Ga-DOTA-Siglec-9

The reaction vessel was preloaded with a mixture of sodium acetate buffer (2.0 mL, 0.2 M, pH 4.0), absolute ethanol (0.2 mL), and precursor DOTA-Siglec-9 (80 µL, 40 µg, 16.5 nmol). [^{68}Ga]$GaCl_3$ was eluted from the generator with HCl (6 mL, 0.1 M) and loaded onto a Strata-XC cation exchange cartridge. The [^{68}Ga]$GaCl_3$ bound to Strata-XC was eluted into the reaction vessel with acidified acetone (0.8 mL, containing 0.02 M HCl and 3.25% water). Before dilution with saline (4 mL, 0.9 mg/mL), the reaction mixture was incubated at 65°C for 6 min. The diluted reaction mixture was passed through a tC18 cartridge, which was washed twice with saline (8 mL, 0.9 mg/mL). Purified [^{68}Ga]Ga-DOTA-Siglec-9 was eluted from tC18 into an end product vial with ethanol (1.3 mL, 70%) via a filter (0.22 µm) and formulated into physiological saline (8.7 mL). The decay-corrected radiochemical yield was 90% ± 3% (n = 3) and radiochemical purity was >95%, as determined by both high-performance liquid chromatography (HPLC) and instant thin layer chromatography (iTLC). Chemical purity was >95% and the total content of DOTA-Siglec-9 peptide in each batch was below the limit of 4.1 µg/mL. The total time for fully automated radiosynthesis was 25 min. The contents of acetone and ethanol in the end product solution were 0.0% (n = 3) and 8.8% ± 0.1% (n = 3), respectively. The product was stable at room temperature for 4 hours (longer times were not tested).

HPLC chromatograms

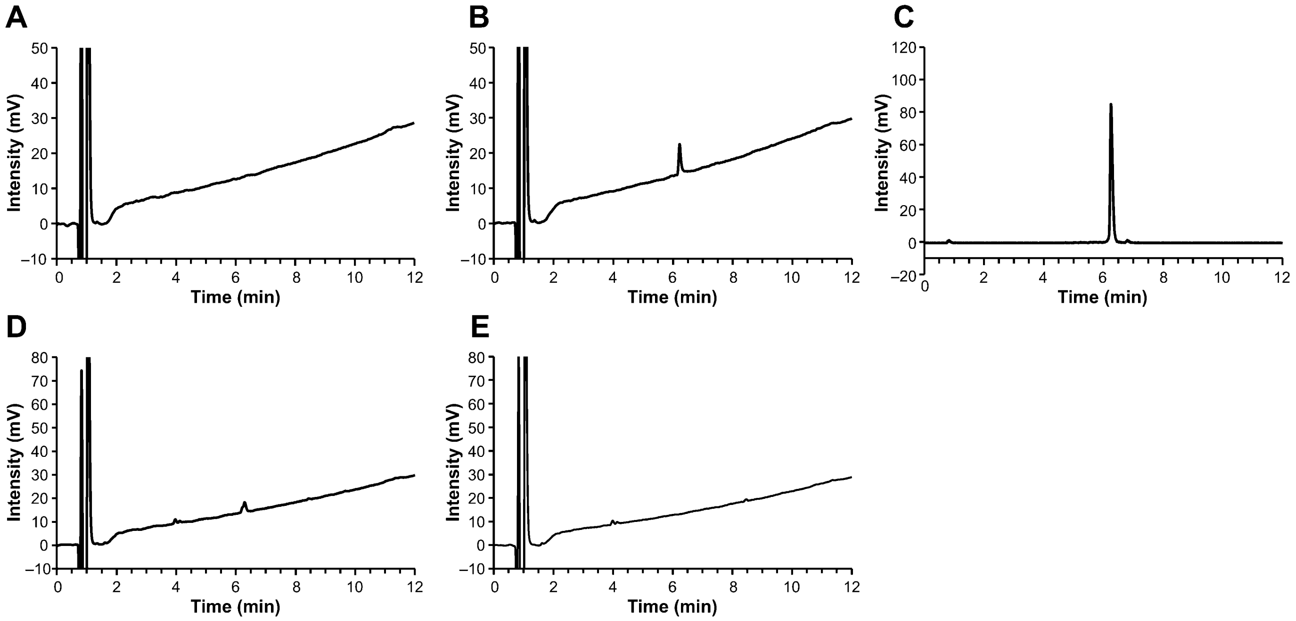

Fig. S1. Quality analysis by HPLC. (A) Blank sample (10% ethanol in saline); UV detection at a wavelength of 220 nm. (B) Reference sample: DOTA-Siglec-9 (4.1 µg/mL); UV detection at a wavelength of 220 nm. (C) [^{68}Ga]Ga-DOTA-Siglec-9; radioactivity detection. (D) [^{68}Ga]Ga-DOTA-Siglec-9; UV detection at a wavelength of 220 nm. (E) A control sample obtained by radiosynthesis in the absence of DOTA-Siglec-9; UV detection at a wavelength of 220 nm. HPLC conditions: Phenomenex Kinetex C18 column, 100 Å, 2.6 µm, 75 × 4.6 mm. Solvent A was water containing 0.16% trifluoroacetic acid (TFA) and solvent B was acetonitrile containing 0.16% TFA. The conditions were as follows: linear gradient from 18% B to 50% B over 12 min; flow rate, 1 mL/min. Retention time was 6.3 min.

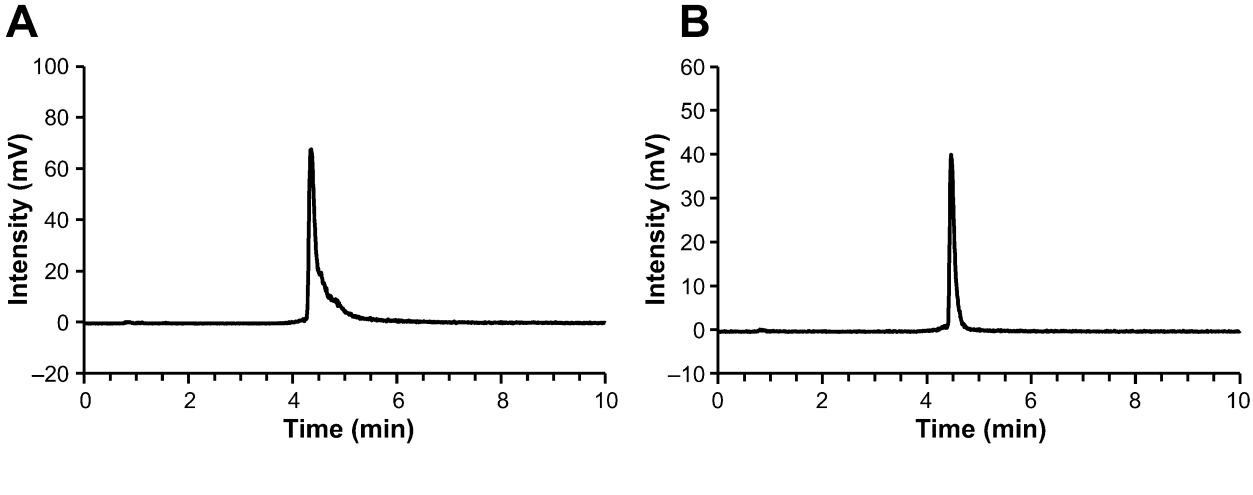

Fig. S2. HPLC analysis of [^{68}Ga]Ga-DOTA-Siglec-9 with different concentrations of TFA in the HPLC solvents and under radioactivity detection. (A) [^{68}Ga]Ga-DOTA-Siglec-9 analysis, with 0.10% TFA in the HPLC solvents. (B) [^{68}Ga]Ga-DOTA-Siglec-9 analysis, with 0.20% TFA in the HPLC

solvents. HPLC conditions: Phenomenex Kinetex C18 column, 100 Å, 2.6 µm, 75 × 4.6 mm. Solvent A was water containing 0.10% or 0.20% TFA, and solvent B was acetonitrile containing 0.10% or 0.20% TFA. Gradient protocol was 18% B from 0 to 1.0 min, from 18% B to 60% B from 1.0 to 5.5 min, 60% B from 5.5 to 7.0 min, from 60% B to 18% B from 7.0 to 7.5 min, and 18% B from 7.5 to 10.0 min. The flow rate was 1 mL/min. Retention time was 4.5 min.

PET studies in rats

Twenty-four hours before the positron emission tomography (PET) studies, Sprague-Dawley rats (n = 3, weight 110 ± 5.9 g) were subcutaneously injected with turpentine oil (Sigma-Aldrich) to induce focal acute, sterile inflammation in right foreleg. Rats had *ad libitum* access to food and water before PET/computed tomography (CT) imaging. The rats were anesthetized with isoflurane and the tail vein was cannulated. CT was performed for anatomical reference and attenuation correction. Then, rats were intravenously (i.v.) administered with [^{68}Ga]Ga-DOTA-Siglec-9 (16.0 ± 6.0 MBq) and a 60-min dynamic PET acquisition was performed (Inveon Multimodality PET/CT, Siemens Medical Solutions, Knoxville, TN, USA). The PET data were reconstructed into 6 × 10 s, 4 × 60 s, 5 × 300 s and 3 × 600 s time frames using an ordered-subsets expectation maximization 3D algorithm. Quantitative PET image analysis was performed by defining regions of interest (ROIs) within the inflamed area (on the right foreleg), control area (on the intact left foreleg), kidneys, heart left ventricle, liver, muscle and urinary bladder using the Inveon Research Workplace software (Siemens Medical Solutions, Knoxville, TN, USA). Results were expressed as standardized uptake values (SUV) and time-activity curves. SUV was calculated as a ratio of tissue radioactivity concentration (Bq/mL) and administered radioactivity dose (Bq) divided by animal's body weight.
Immediatelly after the PET imaging, rats were sacrificed and various tissues were excised and weighed, and their radioactivity levels were measured with a gamma counter (Triathler 3", Hidex Oy, Turku, Finland). The *ex vivo* radioactivity measurements were corrected for radionuclide decay from the time of injection and the weight of tissue and animal, and the results were expressed as percentage of injected radioactivity dose per gram of tissue (%ID/g).

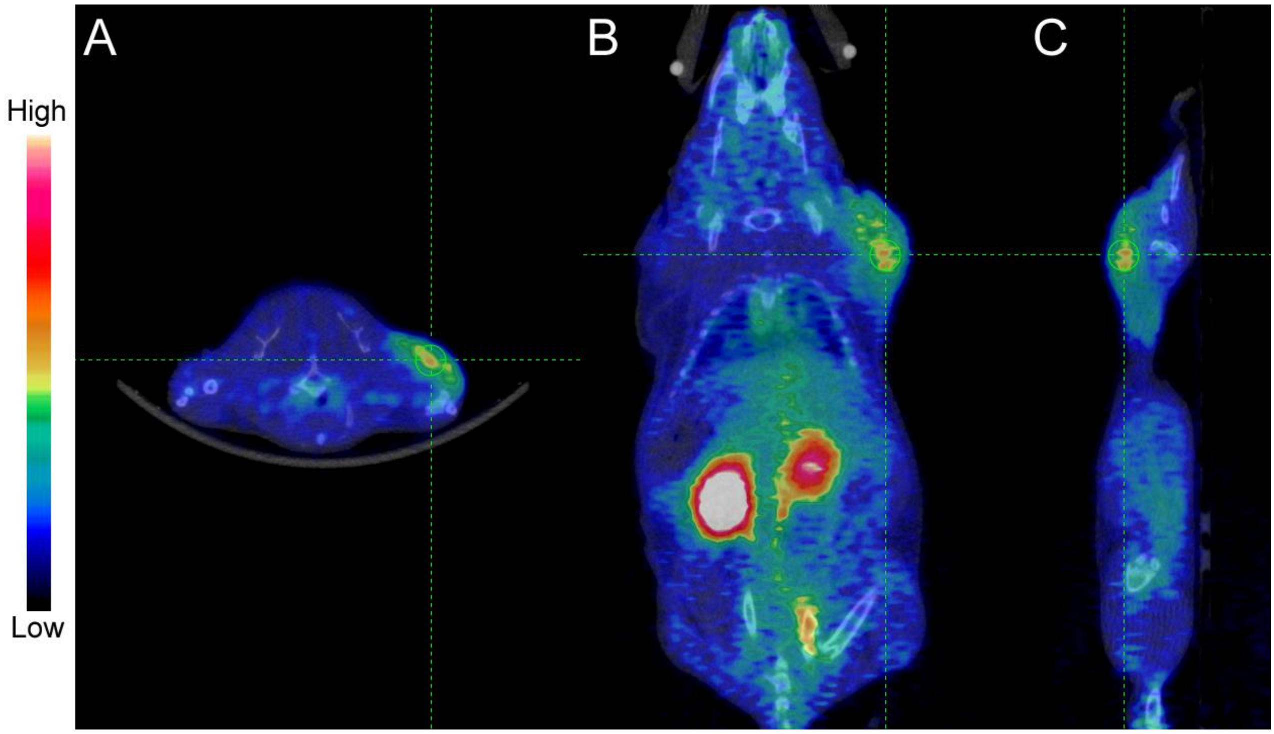

Fig. S3. Representative (A) transaxial, (B) coronal and (C) sagittal [^{68}Ga]Ga-DOTA-Siglec-9 PET/CT images of a rat with turpentine-induced skin inflammation (marked region).

Table S1. *Ex vivo* biodistribution of *i.v* injected GMP grade [^{68}Ga]Ga-DOTA-Siglec-9 in rats at 60 min post-injection.

Tissue	Mean ± SD (*n* = 3)
Inflamed area	0.48 ± 0.12
Control area	0.25 ± 0.072
Blood	0.47 ± 0.070
Plasma	0.76 ± 0.13
Muscle	0.10 ± 0.023
BAT	0.14 ± 0.036
WAT	0.11 ± 0.034
Bone (femoral bone+marrow)	0.30 ± 0.031
Bone (skull)	0.21 ± 0.019
Brain	0.023 ± 0.005
Heart	0.17 ± 0.018
Kidneys	3.7 ± 1.4
Liver	0.35 ± 0.056
Lungs	0.28 ± 0.052
Pancreas	0.13 ± 0.025
Small intestine	0.20 ± 0.027
Spleen	0.15 ± 0.0039
Urine	97 ± 18

Results are expressed as percentage of injected radioactivity dose per gram of tissue (%ID/g, mean ± standard deviation); BAT = brown adipose tissue; WAT = white adipose tissue.
